# Job descriptions as drivers for changes in practice patterns: a cross-sectional survey of Nurse Practitioners in Norway

**DOI:** 10.1186/s12912-025-03747-w

**Published:** 2025-08-19

**Authors:** Else Turid Pedersen, Mette Tøien, Lisbeth Fagerström, Linn Hege Førsund

**Affiliations:** 1 Department of Nursing and Health Sciences, Faculty of Health and Social Sciences, University of South-Eastern Norway, Drammen, Norway; 2 Department of Natural and Health Sciences, Faculty of Natural Sciences and Engineering, Åbo Akademi University, Turku, Finland; 3 University of South-Eastern Norway, PB 4, 3199, Borre, Norway

**Keywords:** Nurse practitioner, Advanced practice nurse, Job description, Practice patterns, Quantitative study

## Abstract

**Background:**

Nurse Practitioners (NPs) are pivotal in transforming healthcare by bridging gaps in delivery and improving patient outcomes through advanced clinical expertise. Internationally, NPs often have clear job descriptions and significant autonomy. However, in Norway, the lack of standardized role definitions creates challenges. This study explores the integration of NPs into the Norwegian healthcare system, highlighting variations in practice patterns due to limited regulatory clarity and standardized job descriptions. Establishing national regulatory mechanisms is essential for the consistent development and integration of NPs, ultimately enhancing healthcare service quality.

**Aim:**

This study aims to explore the demographic and professional characteristics of NPs in Norway and analyse the influencing factors on changes in their practice patterns following the completion of NP education.

**Methods:**

This study utilized a quantitative, cross-sectional design to collect data over a four-month period, from mid-October 2023 to mid-February 2024. Data analysis was performed using Stata^®^ 18.0, focusing on demographic, education, work condition, and changes in practice patterns following NP education, and specialist approval. Categorical variables were presented as frequencies with proportions, while continuous variables were presented as means with range. Logistic regression was used to examine associations between changes in practice patterns and factors such as workplace, graduation time, and job description, employing both univariable and multivariable analyses.

**Results:**

The sample included 95 participants, the majority of whom were educated in the South-Eastern Norway, and 74% had obtained specialist approval. The results show that 66% of participants reported changes in practice patterns after completing NP education, but only 26% had formal job descriptions. Statistical analysis revealed a strong association between having job descriptions and changes in practice patterns (OR 9.5, *p* = 0.005). Those who graduated after 2020 were less likely to report changes compared to those who had graduated earlier (OR 0.18, *p* = 0.016).

**Conclusion:**

Job descriptions are critical drivers for changes in practice patterns among NPs, but few participants reported having such descriptions. This underscores the need for the healthcare system to assess and tailor NP job descriptions to meet its needs and maximize competency utilization.

## Background

Nurse Practitioners (NPs) are transforming healthcare worldwide by bridging gaps in care delivery, addressing critical healthcare workforce shortages, and improving patient outcomes through their advanced clinical expertise and holistic approach to care [1, 2]. Clearly defined job descriptions are essential to empower NPs to fully utilize their competencies, collaborate effectively within interdisciplinary teams, and contribute to high-quality patient care [3]. However, the scope of NP practice varies significantly across countries, and is influenced by diverse national regulations, healthcare structures, and institutional policies [1, 4]. This challenge is also present in Norway and other Nordic countries, where the NP role is relatively new and still developing within the healthcare system. Internationally, NPs are recognized as autonomous clinicians who can diagnose, treat, and manage healthcare independently or in collaboration with physicians across primary and specialized healthcare services, often with the authority to prescribe medications. The level of practice autonomy and accountability is shaped by the specific context of each country and healthcare setting [2]. In countries where NP roles are most advanced, such as the United States, Canada, Australia, and the United Kingdom, NPs often have clearly defined responsibilities, greater autonomy, and clear job descriptions that facilitate the full utilization of their competencies [5–8]. In contrast, countries that are still in the progress of integrating NPs face challenges such as unclear job descriptions, leading to role ambiguity, limited professional recognition, and barriers to effective practice [4]. The absence of formal job descriptions inhibits the integration of NPs into healthcare systems and causes delays in their effective implementation. Establishing formal job descriptions can enhance both the quality of patient care and the operational efficiency of healthcare services [3, 4].

Over the past decade, Norway has introduced the NP role into its healthcare system, with a primary focus on primary healthcare, to address the growing demand for advanced nursing expertise [9, 10]. However, a lack of standardized job descriptions and regulatory clarity has led to variations in how NPs are utilized across different healthcare settings. Unlike countries where NPs have full practice authority [1], Norwegian NPs often operate within existing regulations as Registered Nurses (RNs) with limited autonomy. This raises concerns about role ambiguity, inconsistent expectations, and barriers to achieving full professional integration [11].

Existing international research has examined practice patterns to understand how NPs approach their roles, and the factors that influence their responsibilities [12–14]. In this study, practice patterns specifically refer to the clinical tasks and responsibilities that NPs in Norway perform as part of their daily work, and how these evolve over time and in response to organizational and regulatory context. These patterns may encompass clinical assessments, diagnostics, treatment and follow-up, triage, communication with patients and families, referrals, coordination of care, as well as, habits, tendencies, decision-making processes, task-shifting, and interactions with other healthcare personnel and patients [12]. Although job descriptions are not a legal requirement, they play an important role in clarifying job characteristics and ensuring consistency in role expectations [15], especially when the role is unknown among patients, colleagues including physicians, and leaders [16].

Internationally, establishing national regulatory mechanisms is recognized as essential for ensuring the consistent development of the NP profession, anchored in a standardized level of competence [2]. Although national regulations for NP education and specialist approval were introduced in Norway in 2020, this recognition does not yet grant expanded clinical authority. Additionally, a nationally standardized framework for job descriptions remains absent. This lack of standardization leads to inconsistencies in how NPs are integrated into healthcare services and recognized by colleagues and employers. It also limits their ability to fully utilize their competencies in clinical practice. This lack of standardization not only complicates NP integration and recognition but also raises questions about how their practice patterns evolve over time. Without clear job descriptions, the extent to which NPs can adapt their responsibilities, shift tasks, and fully apply their advanced competencies remains uncertain [3]. Understanding these dynamics in the Norwegian context is critical for optimizing NPs’ integration into healthcare systems and ensuring their competencies are effectively utilized.

## Methods

### Aim of the study

The aims of this study are to explore the demographic and professional characteristics of NPs in Norway and to analyse the factors that influence changes in their practice patterns following the completion of NP education. Practice patterns in this context refers to the scope, type, and frequency of clinical tasks and responsibilities performed by NPs—such as assessment, diagnosis, treatment, follow-up, and care coordination—and how these have evolved in relation to their advanced training, role development, and organizational and regulatory contexts.

## Study design and setting

This study utilized a quantitative, cross-sectional design to collect data over a four-month period, from mid-October 2023 to mid-February 2024. A cross-sectional design enables the examination of relationships between different variables within a population at a specific point in time [17].

## Sample and data collection

The study sample consisted of NPs who had graduated from master’s programmes in advanced practice nursing in Norway. These participants represent graduates from the first programme established in 2011, reflecting the evolution to six university colleges and universities offering the programme by 2024. Due to the lack of a national registry of NPs holding a master’s degree in advanced practice nursing, we conducted a comprehensive mapping to identify the population of NP graduates from Norwegian university colleges and universities. By February 2024, this mapping process had identified a total population of 237 NPs, of whom 153 held specialist approval as recognized by the National Health Directorate.

The study was launched at the National Advanced Practice Nurse Conference in mid-October 2023, where brochures were distributed to attendees. These brochures included detailed information about the study and a direct link to the questionnaire hosted on Nettskjema^®^, which is a Norwegian digital platform for surveys and registrations. At the same time, an information letter and a direct link to the questionnaire were shared through closed NP groups on social media. Despite these efforts, the initial response rate was low, with only 20 responses received within the first two weeks. To address this, the study outreach was expanded to include members of the professional NP group in the Norwegian Nurse Association, and the invitation was also shared openly on social media. This resulted in an additional 39 responses over the following three weeks. To further boost participation, the survey was again promoted through social media and with support from the professional NP group in the Norwegian Nurse Association. The data collection period ended in mid-February 2024, yielding a total of 101 responses. Six responses were from individuals who were still students, and these were therefore excluded from the study.

## Questionnaire

Data were gathered using an online questionnaire that was based on a modified version of the “Finnish Nurse Prescriber Questionnaire” (FinNPQ) [18]. The FinNPO questionnaire was developed from validated instruments exploring advanced clinical roles [12, 19], nurse prescribing [20, 21], and job satisfaction [12, 22]. The content domains draw on the Caring Advanced Practice Nurse Model [23, 24] and existing empirical work [19, 25, 26]. The questionnaire was reviewed and pilot tested by a national expert in nurse prescribing and nurse prescribers from different healthcare contexts, who provided feedback on content and structure [18]. In this study, we included the items covering demographic information (gender and age), educational background (educational institutions, postgraduate education, and years of experience as RN prior to starting NP education), work conditions (employment percentage, employment type, and workplace), and professional characteristics (changed practice patterns after NP education, formal written job description, and specialist approval). Some variables had highly detailed response options. To ensure participants’ anonymity, these variables were recategorized into broader categories. The year of NP education completion was dichotomized into two categories: up to 2019 (inclusive) and 2020 onwards, to align with the implementation of national regulations in 2020 that formally established the NP role in Norway. Work region was dichotomized into “South-Eastern Norway” and “Other Regions in Norway”. Changes in practice patterns following NP education were dichotomized as “Yes/Partly” and “No”. The remaining part of answers of the FinNPQ will be used in another study regarding a more detailed study of Norwegian NPs’ practice patterns.

### Statistical analysis

Data analysis was performed using Stata^®^ 18.0, SE-Standard Edition for Windows. Descriptive analyses of all participants were performed for demographic, education, work condition, and changes in practice patterns following NP education, and specialist approval. Categorical variables were presented as frequencies with proportions, and continuous variables as means with range [27].

In the analyses of changes in practice patterns, participants with missing workplace information (*n* = 1) and those participants who only worked in education sector (*n* = 4) were omitted from the analyses, as they do not engage in direct patient care. These exclusions were necessary to ensure that our analysis accurately reflected the experiences and patterns of NPs involved in direct patient care.

As some participants reported dual work engagements, their main employment was identified and categorized as either specialist health services or primary healthcare, based on their responses to subsequent questionnaire items. This categorization was crucial for distinguishing between different healthcare settings and understanding specific practice patterns. For one participant, categorization was not possible (marked as missing). Therefore, 89 of the 95 participants, all involved in direct patient care, were included in the subsequent regression analyses.

Logistic regression analyses were performed to study the associations between changes in practice patterns following NP education and factors such as workplace, time of graduation, and job description. Both univariable and multivariable analyses were performed. Results are presented as odds ratios (ORs) together with 95% confidence intervals (CIs) and p-values [27].

### Ethical considerations

The study was approved by the Norwegian Agency for Shared Services in Education and Research (SIKT) (project number 529225). Participants were informed that submitting the completed questionnaire constituted their provision of consent to participate in the study.

## Results

### Participants

The final sample included 95 individuals, representing a response rate of 40% of a total population of 237 NPs at that time. The average age of the participants was 41 years. A substantial majority (94%) had pursued their education from Norwegian university colleges or universities located in the south-eastern region of Norway. Two-thirds of the participants had not pursued any postgraduate education prior to enrolling on the NP education. On average, participants had acquired 2.5 years of nursing experience before beginning their NP education. Of the participants, 27% had completed their NP education before the implementation of the new regulative framework in 2020. Additionally, 74% had obtained specialist approval (see Table 1).

Geographically, most participants (82%) were employed in the south-eastern region of Norway. The majority (92%) held a permanent position and most (90%) worked full-time. Following completion of their NP education, 66% reported a complete or partial change in their practice patterns. However, only 26% reported having a formal job description (see Table 1).

**Table 1:** Demographic and professional characteristics of Nurse Practitioners (NPs), *n* = 95

Variable	Level	*n*	%*
Gender	Female	88	92.6
	Male	7	7.4
Age, years	Mean (range)	95	41.3 (26–62)
Educational institution	Inland Norway University of Applied Sciences	12	12.6
	University of Oslo	19	20.0
	University of South-Eastern Norway	28	29.5
	Lovisenberg Diaconal University College	21	22.1
	Østfold University College	9	9.5
	Other educational institution	6	6.3
Experience as RN before NP education, years	Mean (range)	95	2.5 (0–16)
Year of NP graduation	Up to 2019 (incl.)	26	27.4
	From 2020 onwards	69	72.6
Postgraduate education before NP education	Yes	33	34.7
	No	62	65.3
Employment status	Permanent employee	87	91.6
	Temporary employee	8	8.4
Employment percentage	Full-time	85	89.5
	Part-time	10	10.5
Region of work	South-eastern Norway	78	82.1
	Other regions of Norway	17	17.9
Changed practice patterns following NP education	Yes/Partly	63	66.3
	No	32	33.7
Job description	Yes	25	26.3
	No	70	73.7
Specialist approval	Yes	70	73.7
	No	25	26.3

*data are presented as numbers and percentages, unless otherwise specified

### Workplace distribution

Participants were employed across three main sectors: primary healthcare services, specialist health services, and the education sector. The majority of participants, 65.3% (*n* = 62), worked exclusively in primary healthcare, followed by 16.9% (*n* = 16) who worked solely in specialist health services. A smaller proportion, 4.2% (*n* = 4), were employed in the education sector only. Moreover, 12.6% (*n* = 12) of the participants reported holding dual employment across multiple workplace sectors, indicating a more diverse employment pattern.

Within primary healthcare settings, participants were broadly distributed across various services, including home care services, primary healthcare institutions, out-of-hours services, general practitioners’ offices, and other daytime services. Some participants held multiple roles, working across different settings within the primary healthcare sector. Notably, 19% (*n* = 18) were employed in designated NP services within primary healthcare.

In the specialist health service, the participants worked in various settings, such as emergency departments, ward departments, outpatient clinics, the Emergency Medical Communication Centre (EMCC), and ambulance services.

### Factors influencing changes in practice patterns

Among participants with a job description, 92% reported changes in practice patterns after completing the NP education, compared to 55% of participants without a job description in their current position. The association remained statistically significant after adjustment for workplace and time of graduation (OR 9.5, 95% CI 2.0–45.5, p-value = 0.005) (see Table 2).

In the unadjusted analysis, we found no differences in the proportion of participants reporting changes in practice patterns after completing NP education between participants working in primary healthcare (60.0%) and those in specialist health services (66.7%). The results remained the same after adjustment for job description and time of graduation (OR 0.94, 95% CI 0.4–4.0, p-value = 0.69) (see Table 2).

Participants who graduated after 2020 were significantly less likely (67.2%) to report changes in practice patterns compared to those who graduated in 2019 or earlier (90.3%). The adjusted odds ratio for this association was 0.18 (95% CI 0.05–0.73, p-value = 0.016) (see Table 2).

**Table 2:** Logistic regression analyses of changed practice patterns, *n* = 89

		Changed practice patterns		Unadjusted			Adjusted		
		No, n (%)	Yes, n (%)	OR	95% CI	p-value	OR	95% CI	p-value
Job description	No	29 (44.62)	36 (55.38)						
	Yes	2 (8.33)	22 (91.67)	8.86	1.9–40.8	0.005	9.56	2.0–45.5	0.005
Workplace	SHS	8 (40.0)	12 (60.0)						
	PHC	23 (33.3)	46 (66.7)	1.33	0.48–3.72	0.582	1.26	0.40–4.0	0.693
Graduation time	Up to 2019 (incl.)	3 (9.7)	28 (90.3)						
	After 2020	19 (32.8)	39 (67.2)	0.22	0.06–0.82	0.024	0.18	0.05–0.73	0.016

OR: Odds Ratio

CI: Confidence Interval

SHS: Specialist Health Service

PHC: Primary Healthcare

## Discussion

The aims of this study were to explore the demographic and professional characteristics of NPs in Norway and analyse the factors that influence changes in their practice patterns following the completion of NP education. The main findings show that having a formal job description is a significant factor that influences changes in practice patterns among NPs. Additionally, the findings reveal that most NPs are employed in primary healthcare and are concentrated in the south-eastern region of Norway. It is noteworthy that the workplace appeared to have a minimal impact on whether NPs reported changes in practice patterns. Furthermore, NPs who completed their education in or after 2020 were significantly less likely to report changes in practice patterns compared to those educated before this time.

The significant association found between having a formal job description and the likelihood of NPs changing their practice patterns underscores the importance of role clarity for effectively utilizing NP competencies. This importance is further emphasized by Van Hecke et al. [28], who highlight that clear competency frameworks and job descriptions are crucial for role clarification, enabling advanced practice nurses to better align their roles with their specialized training and competencies. Such frameworks not only support personal and professional development but also guide practice and promote integration into healthcare systems [28]. Behrens [29] also argues that formal job descriptions are essential for the successful implementation of the NP role, as they provide clarity regarding role expectations and responsibilities. This clarity might support NPs to better understand and adjust to their scope of practice, thereby promoting consistent application of their competencies. Additionally, well-defined job descriptions contribute to the shape of professional identity, guide practice, and promote integration into the healthcare system. According to Goulart et al. [30], job descriptions not only outline tasks and responsibilities but also play a crucial role in recruitment by attracting and selecting suitable candidates and establishing clear expectations. Furthermore, formal job descriptions might increase employee motivation and satisfaction, help address conflicts amongst colleagues, and support the development of employee potential [3]. Given the current limitations in legal rights and autonomy for NPs, such as prescription authority, clear job descriptions become even more crucial. They serve as a vital tool to delineate the NP role within the healthcare system, ensuring that NPs can operate effectively within their defined scope despite these constraints.

Henni et al. [31] and Unsworth et al. [32] also highlight the value of role clarity and stress the importance of aligning job descriptions with NPs’ advanced education and competencies. They identify a significant gap between the competencies that NPs possess and their ability to effectively apply them in daily practice. This discrepancy suggests that without well-defined job descriptions that reflect their specialized training, NPs risk underutilizing their competencies, which can hinder professional development and the potential impact of their role in healthcare service. Fried et al. [33] claim that unclear role clarity imposes a high cognitive load on employees, requiring them to continuously expend energy to determine how to perform their tasks and responsibilities, which can reduce both motivation and effectiveness. In contrast, role clarity significantly enhances job performance, improving both individual outcomes and team dynamics [15]. This positive effect of role clarity is not unique to the healthcare sector but has also been observed in other industries; for instance, in the garment industry, role clarity, along with resource availability and employee motivation, has been shown to significantly predict job satisfaction, job performance, and organisational effectiveness [15, 34].

The majority of NPs in this study are employed in primary healthcare. This aligns with the authority’s investment in NP master’s programs through salary subsidies targeted at primary healthcare over several years [35]. These efforts reflect the focus on addressing the demand for NP competencies, which has been identified as being most critical within primary healthcare in many countries [4, 9, 36]. The main reasons for introducing NPs in Norway were to improve access to care and increase the quality of care within the primary healthcare settings [4].

Despite the relatively small sample size, this study reveals a broad distribution of NPs across various areas of primary healthcare and specialist health services. This finding underscores not only the significant role of NPs within primary healthcare but also their versatility and capacity to acclimatize to various healthcare environments. The presence of NPs in multiple settings suggests that the NP role is becoming more established and recognized across the healthcare system. Such a broad workplace distribution aligns with international trends, where NPs are employed across nearly all healthcare settings and in higher education institutions, demonstrating their essential contribution to the healthcare workforce [7]. However, this development also underscores the need for formal job descriptions adapted to diverse settings, ensuring that NPs can fully utilize their competencies in all areas.

This study shows that the workplace itself did not have a significant impact on changes of practice patterns among NPs. This suggests that structural or contextual factors within healthcare settings may be less important than role clarity and recognition. Fatemi et al. [37] point out that individual characteristics, along with organizational, social, cultural, political, and environmental factors, can affect how NPs adjust their practice patterns. A key factor is the lack of awareness and understanding of the NP role, particularly among physicians, nursing leaders, and other healthcare personnel. This lack of understanding can result in insufficient support, which consequently hinders NPs from altering their practice patterns to fully utilize their competencies [38–40]. Without formalized job descriptions and clear communication about the NP role in the entire country, even supportive workplaces may struggle to facilitate meaningful changes in practice patterns. This underscores the need for broader efforts to enhance role awareness and formalize NP competencies across various settings, ensuring NPs can apply their advanced skills regardless of their workplace. This responsibility will be likely to fall on a combination of health service providers, professional nursing organizations, educational institutions, and government health departments. These entities should collaborate to ensure consistency and clarity in the roles and expectations of NPs across the country.

Improving the distribution of NPs across rural areas emerges as a crucial point of discussion in this study. The majority of NPs work in the eastern region of Norway, where population density is highest [41]. This concentration, noted by the OECD [42] as a common trend, underscores the need to attract and retain qualified healthcare personnel in less populated areas [11]. Factors such as the location of educational institutions may influence this uneven distribution [43]. Thus, strategies for decentralized education and incentives for NPs to work in rural regions are necessary. Further complicating the equitable distribution of the healthcare workforce is the common practice in Norway of healthcare personnel holding multiple work engagements, often due to part-time positions in healthcare settings [44]. Several NPs in this study reported having dual work engagements. Addressing this issue to improve healthcare services in areas with lower population density is of paramount importance [42, 45]. Leaders and professional organizations, such as the Norwegian Nurse Association, play a critical role in the dissemination of knowledge and advocating these changes. Drawing a parallel with the influential role that the American Association of Nurse Practitioners has played in the United States [7], the recently established Norwegian Nurse Association’s special interest group of NPs could potentially have a similar influence in shaping the profession and addressing these issues in Norway.

A key observation in this study is that many NPs who graduated after 2020 have not significantly changed their practice patterns. It is unlikely that Norway has educated too many NPs, or that the need for NPs in the country is not substantial. Instead, it is more plausible that challenges exist in implementing the NP role within the healthcare system. This pattern may underscore ongoing difficulties in fully integrating NPs and maximizing their roles within the system. The slow progression may be attributed to systemic barriers, such as insufficient support structures and limited recognition of the NP role among healthcare professionals. Additionally, although Norwegian NPs have achieved specialist approval, this does not grant them expanded rights, such as prescription authority or referral rights. This regulatory limitation may restrict their ability to apply their advanced competencies, thereby contributing to the lack of change in practice patterns observed among recent graduates. Unsworth et al. [32] and Torrens et al. [40] have identified a potential trend of NPs reverting to traditional roles, which may indicate a gap between the demand for NPs’ expertise and the practical application of their competencies [31, 32]. Addressing the slow development of the NP role requires prioritizing strategies that support NPs and foster collaboration, rather than allowing barriers to dominate the conversation [40]. Zhao et al. [46] and Rossiter et al. [47] claim that support from management, staff, and colleagues is vital for the sustainable development of NP roles. They argue that establishing robust support structures involves developing sustainable funding models, providing adequate physical resources, ensuring robust information infrastructure, and offering mentorship. Moreover, national policies and local implementation efforts must work in tandem to create an environment where NPs can thrive and fully utilize their competencies [46, 47]. Without such coordinated efforts, the gap between NP education and practice is likely to persist, thereby limiting the potential impact of NPs within the healthcare system. Formalizing extended rights for NPs, such as prescription and referral authority, and clearly incorporating these rights into standardized job descriptions could be a crucial step towards enhancing their professional autonomy. Such formalization would not only improve NPs’ ability to utilize their competencies but also support their integration into various healthcare settings. Additionally, establishing clear job descriptions that reflect these expanded roles could enhance inter- and intraprofessional collaboration and clarify expectations within healthcare teams.

### Limitations of the study

This study had several limitations that should be considered. The low response rate could limit the representativeness of the results, as those who chose to not respond might have provided differing perspectives or experiences [48]. Furthermore, the use of self-reported data introduces potential biases, such as memory errors, exaggeration, and social desirability. This may impact on the accuracy and reliability of the results. The generalizability of the study’s findings may be limited by the fact that most participants were employed in the eastern region of Norway, potentially excluding perspectives from other geographical and academic areas. Additionally, the lack of job descriptions among a large portion of participants may have hindered a comprehensive understanding of how job descriptions influence practice patterns.

## Conclusion

The existence of job descriptions emerges as a critical factor driving changes in practice patterns among NPs in this study. However, very few of the participants reported having such descriptions. This finding highlights an important area of focus for the healthcare services moving forward: assessing the needs of the healthcare system and tailoring the job descriptions of NPs to align with these needs. Currently, there is no formal national framework for NP job descriptions in Norway, and the clinical role of NPs is often shaped at the local level. Establishing structures that support and facilitate innovative task and role distribution is essential to maximize the use of NPs’ competencies. Future research should investigate specific factors that facilitate or hinder the full utilization of NP competencies across different healthcare environments.

## Data Availability

The datasets generated and analysed during the current study are not available for public use, due to confidentiality, but are available from the corresponding author on reasonable request.

## Abbreviations

NP: Nurse practitioner
RN: Registered nurse
OECD: The Organisation for Economic Co-operation and Development
